# With a Little Help from My Friends: The Role of Detergents for Energy, Efficiency, and Hygiene in Domestic Dishwashing

**DOI:** 10.1002/cplu.202400657

**Published:** 2024-11-22

**Authors:** Dirk P. Bockmühl, Thomas J. Tewes

**Affiliations:** ^1^ Faculty of Life Sciences Rhine-Waal University of Applied Sciences Marie-Curie-Str. 1 47533 Kleve Germany

**Keywords:** Domestic dishwashing, Hygiene, Cleaning performance, Detergents

## Abstract

Domestic dishwashers have become an integral part of modern households, offering convenience and effective cleaning. However, efforts to reduce energy and water consumption have resulted in longer wash programmes, which, despite their high cleaning performance, are not well accepted by consumers due to their long durations. While these eco‐programmes are presumed to be well‐optimized in terms of their structural components, the role of detergents has been somewhat overlooked, despite their significant contribution to both the cleaning and hygiene performance of domestic dishwashers. Beyond machine parameters, the chemical composition of detergents is crucial in improving the cleaning, drying and hygiene performance of household dishwashers, as they contain various components ‐ such as surfactants, enzymes, and bleaching agents ‐ that interact with soil residues to improve cleaning effectiveness. This review will focus on recent scientific efforts to optimize programme structures concerning cycle durations and energy consumption and the role of detergents in this interrelationship between cleaning, drying and hygiene.

## Graphical abstract

Beyond machine parameters, the chemical composition of detergents is crucial in improving the cleaning, drying and hygiene performance of household dishwashers. According to Sinner's principle they might also help to improve the energy efficiency by compensating for lower temperatures.

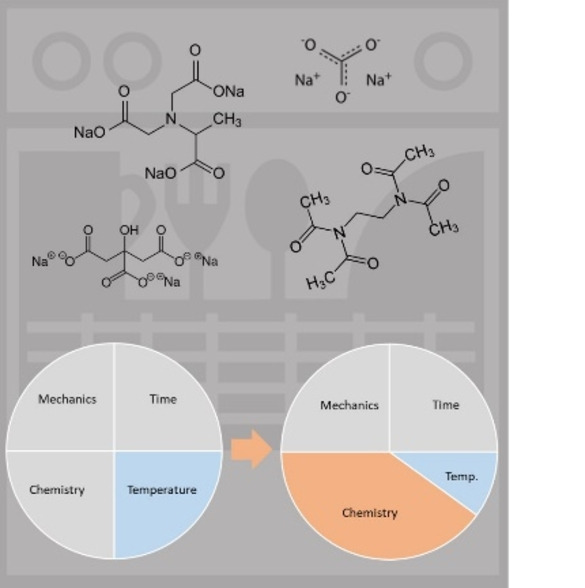

## Introduction

The Sinner's principle, originally introduced in 1960,^[1]^ describes how mechanical action, time, temperature, and chemistry interact in the cleaning process. It also suggests that if one of these factors is adjusted, it can be compensated for by changes in the others. This principle has been used to save energy in devices such as washing machines and dishwashers, leading to programmes that run at lower temperatures but have extended durations. However, while this general principle works well for most devices, it somewhat overlooks an important aspect: other parameters, such as chemistry, are often not considered as compensating factors when temperatures are decreased. This can be understood from the perspective of appliance manufacturers, but needs to be questioned from a holistic approach to achieve maximum efficacy with minimal energy consumption.

In addition to the mechanical and thermal factors that are traditionally emphasized, the chemical composition of detergents plays a crucial role in improving the cleaning effect. Detergents contain various active ingredients that interact with soils at a molecular level. Surfactants, for example, reduce the surface tension of water, allowing it to penetrate fats and oils more effectively and emulsify them. Enzymes break down complex organic substances into simpler compounds that can be easily washed away. The presence of bleaching agents further enhances the cleaning process by oxidizing stains and providing antimicrobial properties. Unlike in laundry detergents, where only solid, universal (but not colour) detergents contain activated oxygen bleach, bleach can be assumed to be present in all solid dishwashing detergents. High‐performance detergents can significantly improve cleaning results, even at lower temperatures, by using advanced chemical formulations that optimize the interaction between detergent components and soil. Understanding the chemistry behind these interactions can help develop more efficient dishwasher programmes. For example, while eco programmes typically run at lower temperatures (around 50 °C) for longer periods of time, research suggests that the addition of specific enzyme blends can achieve comparable cleaning results in shorter cycles. Tewes *et al*. demonstrated that certain combinations of detergents and short cycles can achieve high cleaning efficacy while consuming less energy compared to conventional eco programmes.^[2]^ This underlines the potential of chemical formulations to compensate for lower heat input without compromising on performance. Integrating a chemical focus into the design and operation of dishwashers is crucial for optimizing both cleaning performance and energy efficiency. By fully utilizing detergent chemistry within the framework of Sinner's principle, manufacturers can develop innovative solutions that meet consumer demands for convenience and sustainability while ensuring effective cleaning and hygiene results.

## Energy Efficiency and Cleaning Performance

Eco programmes are designed to effectively clean normally soiled dishes while minimizing energy and water consumption. These programmes typically operate at lower temperatures (around 50 °C) and have longer cycle times (often exceeding 3 hours) than standard programmes like Auto, Intensive or 1 h programmes.[^3^, ^4^] While eco programmes are highly efficient in terms of resource usage, their extended duration has been a concern for many consumers, with surveys indicating that a significant portion find them too long.[^3^, ^5^]

In contrast, short programmes lasting less than 1 hour have gained popularity among consumers seeking convenience.^[3]^ Notably, some short programmes may consume equal or even lower amounts of energy and water compared to eco cycles.[^2^, ^6^] Consequently, Alt *et al*. showed that implementing recommendations for using short programmes for lightly soiled dishes could lead to energy savings of over 20 % and water savings of around 30 %.^[7]^ This finding is supported by a study by Tewes *et al*., suggesting that the detergent used has a major influence on the cleaning result, especially in short cycles with a duration of less than 60 min and a temperature of 45–50 °C during the main wash. The same study demonstrated that certain combinations of short cycles and cleaners achieve similar results to eco cycles.^[6]^ Therefore, it must be assumed that short cycles may be a viable alternative to eco cycles if supported by a strong detergent.

In this context, it's important to note that the industry standard IEC 60436, which tests the cleaning performance of domestic dishwashers, uses a reference dishwashing detergent that is less effective than many common good performing market detergents.[^6^, ^8^] Many detergents, including the IEC reference detergent, primarily contain sodium citrate and a maleic acid/acrylic acid copolymer, with sodium silicate serving as the complexing agents and builder components, respectively (Table 1). However, high‐performance market detergents regularly incorporate a wider range of ingredients, including additional polymers and complexing agents. The quality of the enzymes used, primarily proteases and amylases, can also vary. Furthermore, while the reference detergent employs a standard bleaching system activated by sodium percarbonate and/or tetra acetyl ethylene diamine (TAED), market detergents may contain manganese‐based bleach catalysts.^[9]^ All these differences may contribute to a much better cleaning efficacy of high‐performance market detergents in short cycles compared to the IEC reference detergent.

**Table 1:** Composition of Reference Detergent D according to.^[8]^

Chemical substance	Mass %
Sodium citrate dihydrate	30.0
Maleic acid/acrylic acid copolymer sodium salt	12.0
Sodium disilicate	10.0
Sodium percarbonate	7.0
Tetra acetyl ethylene diamine (TAED)	2.0
Linear fatty alcohol ethoxylate	2.0
Protease	1.0
Amylase	0.5
Sodium carbonate	ad 100

By fully utilizing the chemical aspects of Sinner's principle, it can be demonstrated that high cleaning performance can be achieved in short programmes.^[6]^ This indicates that high‐performance detergents may considerably contribute to energy savings in domestic dishwashing, as their chemistry can efficiently compensate for reductions in appliance parameters such as temperature, time, and water consumption. Indeed, recent data from Tewes *et al*. suggest for example that high‐performance detergents can achieve a cleaning efficacy of up to 92.4 % in short programmes compared to 95.3 % in eco programme, all while consuming significantly less energy.^[6]^

However, these investigations did not consider the drying performance and thus must be viewed with some caution, since more energy might be needed to achieve a satisfying drying result.

Moreover, it should be noted that these investigations were made according to the IKW “Recommendations for the Quality Assessment of the Cleaning Performance of Dishwasher Detergents”^[10]^ and may not be directly comparable to measurements based on the IEC standard.^[8]^ As shown in Table 2, the temperatures for the main cleaning and the rinse phases were chosen to be similar (if not equal) to those of the eco programme, while water consumption and cycle times were considerably reduced. Interestingly, the high‐performance values (stated in Table 2) could only be achieved with a high‐performing market product, whereas the reference detergent performed much weaker in the short cycles. This is expected, as the IEC reference detergent was designed to allow for differentiation of dishwasher performances, which is only possible with a less effective detergent.

**Table 2:** Averaged cleaning performance and energy consumption on IKW soils^[10]^ of selected short programmes compared to an eco programme (taken from^[6]^).

Programme	Short 1	Short 2	Short 3	Eco
T main [°C]	50	50	45	50
Time [min]	40	40	40	197
Water [L]	8	8	9	11
T rinse [C]	50	40	50	50
Energy [kWh]	0.494	0.405	0.484	0.623
Cleaning [%]	93.9	92.4	91.6	95,3

The reference detergent is being updated regularly with a recent decision to move to reference detergent E in 2025. However, it is anticipated that it will continue to exhibit weak performance to maintain its differentiating nature. Although the study from Tewes *et al*.^[6]^ used market detergents to determine cleaning performance, these data should not be regarded as direct recommendations for consumers, since the machine parameters used may not meet the requirements for programmes in commercially available devices. For instance, as mentioned, the drying performance was not assessed in this study. Rather, these data should be seen as guidelines for the future development of shorter programmes that can deliver good cleaning performance with a market detergent, without the long duration of an eco programme.

To achieve this, collaboration between detergent and appliance manufacturers may be necessary, as well as a harmonization of experimental methods. Currently, these methods are divided into standardized scenarios for efficacy testing of detergents (IKW^[10]^) and machine performance (IEC^[8]^).

## Hygiene Performance

While energy efficiency and cleaning performance are often prioritized, the hygiene aspects of dishwasher programmes should not be overlooked. Like the cleaning process, hygiene efficacy in appliances is influenced by the four interdependent factors of the Sinner Circle.^[9]^ Eco programmes, with their longer durations, generally achieve satisfactory hygiene results, even at lower temperatures, as shown by Brands *et al*.^[12]^ A crucial detergent component has proven to be the bleaching system, which has already been shown to have a major influence on the hygiene efficacy of laundering processes, too.^[11]^ In shorter programmes, activated oxygen bleach significantly enhances antimicrobial activity, while the effect is less pronounced in longer programmes. Bleach‐free detergents show good hygiene efficacy at higher temperatures, and in programmes with a cleaning cycle longer than 45 minutes.^[12]^ It thus stands to reason that effectively utilizing Sinner's principle could lead to an optimized hygiene efficacy as well as low water and energy consumption. All Sinner Circle factors should be considered in this regard: time, temperature, chemistry and (to a lesser extent) mechanical action.

Achieving antimicrobial effects in dishwashing can occur in two ways: (1) microbial cells embedded in food residues can be removed physically; a process which resembles a cleaning effect and/or (2) microorganisms can be an inactivated by chemical or thermal means. Consequently, while good cleaning efficacy mostly results in good hygiene efficacy, antimicrobial effects still need to be considered as microbial cells might still be able to adhere to ‐ and survive on ‐ visually clean surfaces. This is particularly true in closed systems like dishwashers where microorganisms can be easily transferred within the device. Taken together, the available data suggests that evaluating the hygiene efficacy of the dishwashing process is more complex than determining cleaning efficacy. Hygiene efficacy in shorter programmes with lower temperatures has not yet been fully addressed and calls for further investigation.

## Predictive Models and Multivariate Relationships

**Figure 1:**
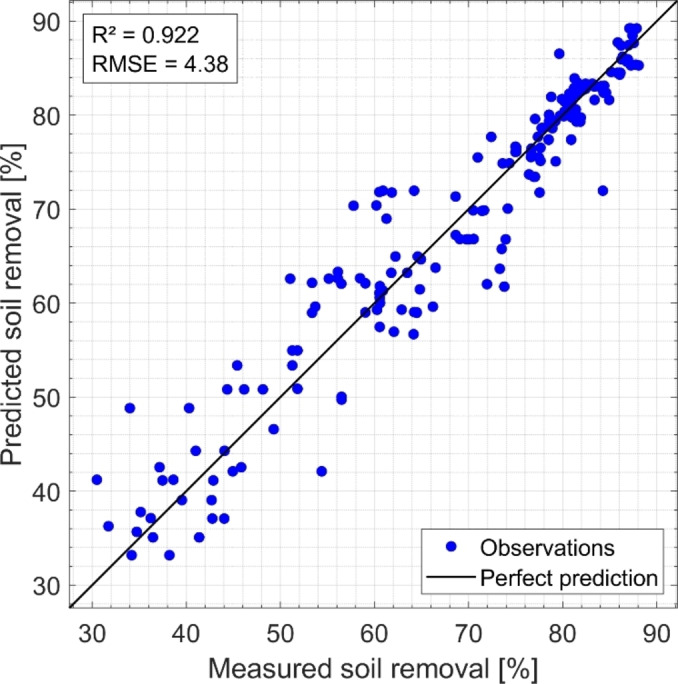
Example of five‐fold cross‐validated predictions for soil removal using a cubic support vector machine based on 11 input variables for prediction.^[2]^ R^2^ (0.922) indicates the high correlation of predicted values with actual values. The relatively low root mean square error (RMSE), indicates an average deviation of +‐4.38 % in the prediction of soil removal. The picture was adapted from^[2]^ published under CC‐BY 4.0: https://creativecommons.org/licenses/by/4.0/.

Predictive models and multivariate analyses provide a means to better understand the complex interplay between programme parameters, cleaners, and cleaning performance. When trained on experimental data, predictive models can forecast cleaning performance for unknown combinations of programme settings and cleaners, taking into account individual soils as well as a generalized performance.^[2]^ Such models provide valuable insights into the multivariate relationships between factors like temperature, time, water volume, and cleaner type, enabling the optimization of dishwasher programmes for both energy efficiency and cleaning efficacy. Figure 1 illustrates the cross‐validated predictions for a defined soil removal in dishwashers. The predictions are based on a support vector machine (SVM) model using almost 200 soil reduction values, which were recorded in laboratory dishwashers, and uses 11 input variables for prediction, including the temperatures of the main wash and rinse cycle, the duration of these phases and water consumption.^[2]^ Four different detergents tested also served as categorical input parameters. The SVM model was trained with a cubic kernel function using the Regression Learner Toolbox of MATLAB R2022b (MathWorks, Natick, MA, USA).^[2]^ To verify the model performance, a five‐fold cross‐validation was performed. In this technique, the data is divided into five equal subsets, the model is trained on four of these subsets and tested on the remaining subset. This process is repeated five times, ensuring that each subset is used once for testing. The average performance across all foldings was used to assess the generalizability of the model and to reduce the risk of overfitting. R^2^ indicates how well the predicted values correlate with the actual values and is high at 0.922, indicating robust model performance. The root mean square error (RMSE) is ideally suited as a measure of the average difference between the predicted and actual values to evaluate the model performance. A low RMSE of 4.38, as in the example shown, means an average expected deviation of ±4.38 % in the prediction of soil removal. Models like this can be used to predict the cleaning performance of new cycles and thus save a lot of development work. Of course, such models must be continually validated and further optimized with new data to meet the relevant requirements.

## Consumer Behavior and Acceptance

Despite the potential benefits of eco programmes, consumer acceptance and behavior play a crucial role in their adoption. Surveys have shown that a significant portion of consumers (~65 % from 11 countries) do not accept longer programmes to save energy.^[3]^ Additionally, many consumers fail to recognize the connection between longer cycle times and lower energy consumption, or even assume that longer cycles consume more energy.^[4]^

Conversely, short programmes act on the demand for convenience and shorter cycle times, with around 18.5 % of consumers in 10 European countries considering short cycle lengths as being important.^[3]^ However, if the cleaning performance of short programmes is suboptimal, consumers may be reluctant to adopt them, or engage in compensatory behaviours, highlighting the need for effective cleaner‐programme combinations.

## Summary and Outlook

In conclusion, both short programmes and eco programmes in domestic dishwashers offer distinct advantages and trade‐offs in terms of energy efficiency and hygiene. While eco programmes are designed for optimal energy efficiency, their extended durations may deter consumer acceptance. Short programmes, on the other hand, offer convenience but their cleaning and drying performance depends on the detergent used and the optimal adaptation of other programme parameters. The scientific literature suggests that certain combinations of short programmes and cleaners can achieve comparable cleaning results to eco programmes while consuming equal or lower amounts of energy and water.[^2^, ^6^] Predictive models and multivariate analysis techniques can aid in optimizing these combinations for specific soils and conditions.

Ultimately, consumer behavior and acceptance play a crucial role in the adoption of energy‐efficient dishwashing practices. It must be assumed that consumers will only adopt sustainable dishwashing habits if they are satisfied with the performance outcome (cleaning, drying and hygiene). Educating consumers about the relationship between programme duration and energy savings, as well as promoting effective cleaner‐programme combinations, could lead to significant reductions in household energy and water consumption.

As the demand for sustainable living continues to grow, further research and innovation in dishwasher technology, coupled with consumer awareness campaigns, will be essential in striking the right balance between energy efficiency, hygiene, and convenience in domestic dishwashing.

Future research should involve closer cooperation between appliance and detergent manufacturers. This could focus on the development of advanced detergent formulations that use innovative chemical compositions to improve cleaning efficiency while minimizing energy consumption. This includes research into novel surfactant and enzyme combinations that achieve high cleaning ‐ drying and hygiene performance even at lower temperatures, as well as the corresponding dishwashers and programmes that satisfy the user in terms of both overall performance and program duration.

## Conflict of Interests

The authors declare no conflict of interest.

1

## Biographical Information

*Prof. Dr. Dirk Bockmühl, studied Biology in Düsseldorf and worked in different positions in the field of laundry and home care in a consumer goods company, before joining Rhine‐Waal University of Applied Sciences in 2010 as a professor for Hygiene and Microbiology. His research interest in in different aspects of non‐clinical hygiene as well as the antimicrobial and cleaning efficacy of detergents, cleaners and appliances*.

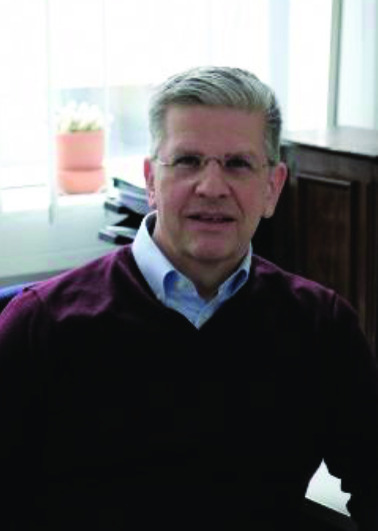

## Biographical Information

*Thomas Tewes M.Sc., Thomas J. Tewes is a research associate at Rhine‐Waal University of Applied Sciences specializing in microbiology and Raman spectroscopy. In his studies, he mainly uses chemometric approaches to analyze multivariate data to investigate microorganisms or to evaluate the cleaning performance and efficiency of household appliances, especially dishwashers. His publications include studies on the effectiveness of different dishwashing parameters and Raman spectroscopic analysis of microorganisms and biofilms on different surfaces*.

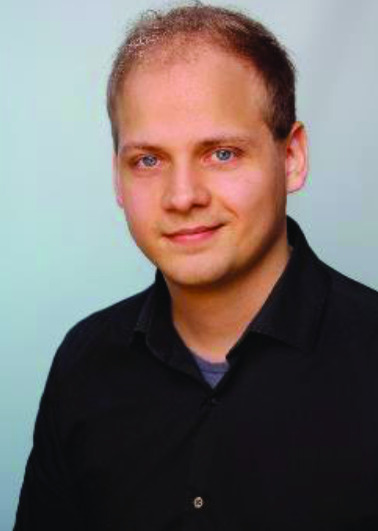

## Data Availability

Data sharing is not applicable to this article as no new data were created or analyzed in this study.
